# Characterization of the Ovine Vaginal Microbiome and Inflammation Patterns as an Improved Testing Model of Human Vaginal Irritation

**DOI:** 10.3389/frph.2021.714829

**Published:** 2021-12-07

**Authors:** Richard B. Pyles, Aaron L. Miller, Carrie Maxwell, Lauren Dawson, Nicola Richardson-Harman, Glenn Swartz, Cynthia O'Neill, Cattlena Walker, Gregg N. Milligan, Timothy Madsen, Massoud Motamedi, Gracie Vargas, Kathleen L. Vincent

**Affiliations:** ^1^Department of Pediatrics, The University of Texas Medical Branch, Galveston, TX, United States; ^2^Office of Clinical Research, The University of Texas Medical Branch, Galveston, TX, United States; ^3^Alpha StatConsult, LLC., Damascus, MD, United States; ^4^Advanced Bioscience Laboratories, Inc, Rockville, MD, United States; ^5^Sinclair Research Center (SRC), Auxvasse, MO, United States; ^6^Department of Ophthalmology and Visual Sciences, The University of Texas Medical Branch, Galveston, TX, United States; ^7^Department of Cell Biology, Neurobiology and Anatomy, The University of Texas Medical Branch, Galveston, TX, United States; ^8^Department of Obstetrics and Gynecology, The University of Texas Medical Branch, Galveston, TX, United States

**Keywords:** vaginal microbiome, ovine sheep vaginal model, toxicity, intravaginal drug delivery, dysbiosis, cytokines, inflammation, women's health

## Abstract

The development of therapies targeted to improve the health of women has utilized direct vaginal delivery as a more effective and less toxic method of protection from HIV and other pathogens. Vaginal applicants and delivery devices that provide sustained effects have been met with increasing acceptability, but the efficacy and toxicity outcomes have not been successfully predicted by preclinical *in vitro* studies and animal modeling. We have explored the utilization of sheep as a model for testing the safety of vaginal applicants and devices based on spatial and structural similarities to the human vagina. As recently noted by the FDA, an additional safety measure is an impact on the vaginal microbiome (VMB) that is known to contribute to vaginal health and influence pathogen susceptibility and drug metabolism. To advance the utility of the sheep vaginal model, we completed a thorough molecular characterization of the ovine VMB utilizing both next-generation sequencing (NGS) and PCR methods. The process also created a custom PCR array to quantify ovine VMB community profiles in an affordable, higher throughput fashion. The results from vaginal swabs (>475 samples) collected from non-pregnant crossbred Dorset and Merino ewes treated with selected vaginal applicants or collected as sham samples established 16 VMB community types (VMB CTs). To associate VMB CTs with eubiosis or dysbiosis, we also completed custom ELISAs for six cytokines identifying IL1B, IL8, TNFa, and CXCL10 as useful markers to support the characterization of ovine vaginal inflammation. The results indicated that *Pasteurella, Actinobacillus, Pseudomonas, Bacteroides, Leptotrichia*, and *E. coli* were common markers of eubiosis (low inflammatory marker expression), and that *Haemophilus, Ureaplasma*, and *Corynebacterium* were associated with dysbiosis (high cytokine levels). Utilizing the optimized workflow, we also confirmed the utility of three commonly used vaginal applicants for impact on the VMB and inflammatory state, producing a dataset that supports the recommendation for the use of sheep for testing of vaginal applicants and devices as part of preclinical pipelines.

## Introduction

The development of vaginally administered drugs and devices utilizes standard preclinical testing methods ranging from *in vitro* testing systems to animal models such as mice, guinea pigs, rabbits, sheep, and non-human primates (NHPs). Strict safety evaluations are required to demonstrate that products do not cause local mucosaltoxicity, tissue injury, and inflammation including impacts on the local microflora. Recent studies have illustrated that the vaginal microbiome (VMB) can have an impact on mucosal health (1–3), susceptibility to infections (3–7), and efficacy and safety of vaginal applicants and devices (5, 8, 9). Clinical findings (10, 11) and early *in vitro* modeling (4, 5) support the need for careful evaluation of the impact of the VMB on the safety and efficacy of vaginal products. It can be concluded that distinct VMBs may impact drug toxicity, drug efficacy, pharmacokinetics, and, because these aspects have been understudied during the preclinical phase, may have helped explain poor clinical trial outcomes.

Preclinical animal studies on vaginal applicants and devices have been limited by the dissimilarity of animal bacterial communities and the human VMB. Each of the commonly used animal models presents distinct microanatomy and mucosal compositions, adding to the distinctions in the microbiota supported by each vaginal environment. Human VMB communities include Lactobacilli that produce lactic acid leading to a reduced pH not found in any current animal models. The human VMB includes five to seven dominant community types that have been associated with eubiosis or dysbiosis based on inflammation markers (12). Few of these models have reported information about the microbiota present in the vaginal cavity. The rabbit vaginal irritation model has served as a standard for decades but is limited by a mucosa lined by columnar epithelium, which ineffectively models the stratified squamous epithelium in humans. Furthermore, the smaller vaginal cavity size of rabbits, as well as other animals, limits sampling consistency and requires that intravaginal product delivery or associated delivery device be modified for preclinical testing (13, 14).

Sheep are less expensive, more easily handled and sampled, and can be purchased in larger numbers than non-human primates (NHPs) based on reduced demand for preclinical studies. The ovine cervicovaginal tract has many anatomical similarities to humans and is of comparable size, allowing for direct testing of vaginal devices designed for human placement without modification. Although the ovine vaginal cavity is slightly smaller than that of a human, it can accommodate human-sized intravaginal rings (IVRs) and other devices, providing an advantage over other models such as the NHP and rabbit (15). The vaginal epithelium in sheep is stratified squamous overlying lamina propria as is found in women, however, the sheep epithelium (50–100 um) is thinner than in reproductive age women (200–300 um), while it is close in thickness to that of postmenopausal women who are not using hormonal replacement (80–110 um). The sheep vagina has a sphincter just proximal to the urethra and, therefore, can hold gels and rings in place. Further, the suitability of sheep as a large animal model for evaluation and testing of the performance of human size intravaginal devices including vaginal rings for sustained delivery of emerging non-vaccine biological preventatives, (e.g. tenofovir), as well as application of several OTC and experimental compounds is indicated by recent reports (15–25). Finally, in addition to standard histopathology of tissues after euthanasia, there are now refined repeated measure methods for ovine vaginal irritation inspection, namely, optical coherence tomography (OCT), colposcopy, and tissue sampling by repeat biopsy.

The FDA acknowledged that the sheep model is relevant for the evaluation of local mucosal toxicity and pharmacokinetic profiling of systemic exposure including the measurement of drug concentrations in vaginal fluid and cervicovaginal tissue. FDA provided guidance on future assessments of vaginally administered HIV prevention products utilizing the sheep model, establishing the necessity for an assessment of effects on the vaginal microflora in safety evaluation studies on sheep (26, 27). Advances have supported the utility of the sheep as a safety model for vaginal injury and local immune responses based on past studies to quantify inflammatory responses induced by the application of irritants such as nonoxynol-9 (N9) and benzalkonium chloride (BZK). Vaginal application of N9 or BZK resulted in foci of disrupted epithelium detectable by confocal endomicroscopy (17, 22). Such treatments also recruited leukocytes to the vaginal mucosa that were enumerated by differential staining and flow cytometry (28). In those reported studies, immune cell populations were similar in sheep to those seen in humans, and a population of predominantly granulocytes and monocytes infiltrated the vagina by 18 h post-treatment, persisting through at least 44 h (28). Similarly, customized quantitative ELISAs were created to quantify proinflammatory cytokines and chemokines in cervicovaginal lavages, showing consistent significant increases in both IL-8 and IL-1β after microbicide application, suggesting that these cytokines, similar to those in humans, were potential biomarkers for epithelial injury (28). The findings supported that sheep vaginal modeling mirrored results obtained from other animal models and human trials with N9, but several additional barriers existed for the full utility of this preclinical testing system.

Although there are limited reports on the sheep VMB that collectively indicate that it is different than that of humans (29–33), a change in the microbiome after use of a drug may be indicative of drug toxicity and should serve as an effective surrogate of impact on the microbiota. In addition, correlating these changes in the VMB to cytokine biomarkers for mucosal injury strengthens their utility for understanding the potential of a given product to cause harm. Current literature regarding the sheep VMB is minimal and is generally focused on the effects of pathogenic organisms on agricultural fertility (30–33). To address this limitation and build on the strength of prior safety studies discussed above, we completed detailed studies of baseline VMB in healthy cycling female ewes. These results were compared to VMB profiles produced after administration of the standard vehicle control hydroxyethylcellulose (HEC) universal placebo gel (34) and known irritant applicants selected to elicit inflammation and potential VMB dysbiosis. Definitions of biosis were based on novel customized ELISA methods to quantify selected cytokines, allowing us to relate ovine VMBs to vaginal inflammatory markers to create both a set of tools and methods to support this aspect of the model, as well as a research workflow that could be employed to develop similar tools and methods for other animal models.

Our workflow exported refined techniques for the sheep model established at the University of Texas Medical Branch (UTMB) to the contract research organization Sinclair Research Center (SRC). Importantly, the successful transference of these techniques and consistency in outcomes from the study demonstrate the utility of the sheep model in a real-world setting used by product developers under good laboratory practices. Using established quality cutoffs, vaginal sample collection was standardized through training, producing a robust dataset for analyses. In a subsequent study performed by SRC using optimized methods, four groups of animals received a vaginal application of either reference standard 4% nonoxynol-9 (N9) contraceptive gel (Conceptrol™), a known chemical vaginal irritant 0.2% benzalkonium chloride (BZK) solution, the standard HEC gel vehicle control, or no applicant. Our three-phase approach completed over 8 years utilized both next-generation sequencing (NGS) and quantitative PCR (qPCR) methods. Collectively, the findings in two breeds of sheep housed under different laboratory animal medicine conditions established 16 common VMB community types (CTs). Following the quantification of selected cytokines (IL-1b, IL-8, IL-6, IL17-a, TNF-a, and CXCL10), the established VMB CTs were assigned to an inflammatory state to distinguish eubitoic and dysbiotic profiles. The information gained from these studies has provided foundational data regarding the impact of known irritants on vaginal microbiota in the sheep. Despite substantial differences to the human VMB, the data associate specific ovine vaginal organisms with dysbiosis, helping advance the utility of the model to predict vaginal environment impacts of applicants or devices prior to clinical studies. The data also established optimized criteria for assessing the preclinical safety of vaginal applicants and devices with respect to predicted impact on the VMB using more sophisticated and customized qPCR arrays.

## Materials and Methods

### Animal Care and Use

Animal studies conformed to the Guide for the Care and Use of Laboratory Animals. All the studies were completed with full IACUC approval from UTMB (Merino sheep studies) or Sinclair Research Center (SRC; Dorset sheep studies). All the animals were provided continuous veterinary care, unlimited access to food and water, and were treated humanely. The animals were observed daily throughout the studies. Field-born Merino sheep were housed at UTMB in an environmentally conditioned space with a 12-h light dark cycle. The studies were completed throughout the year. The caging had slotted flooring that was washed automatically every 8h. In contrast, field-born Dorset ewes were maintained in a partially open-air shelter and fed a combination of pelleted grasses and unprocessed hay that was in direct contact with the flooring. Estrus cycles were not measured for any of the animals. The Dorset animals were all studied in the summer months but bred throughout the year including during normal anestrus periods for other breeds. UTMB housing conditions likely impacted seasonal anesthrus due to regular light dark cycles because ovulation was observed during months of typical anestrus.

### Study Design and Application of Compounds

**Figure 1:**
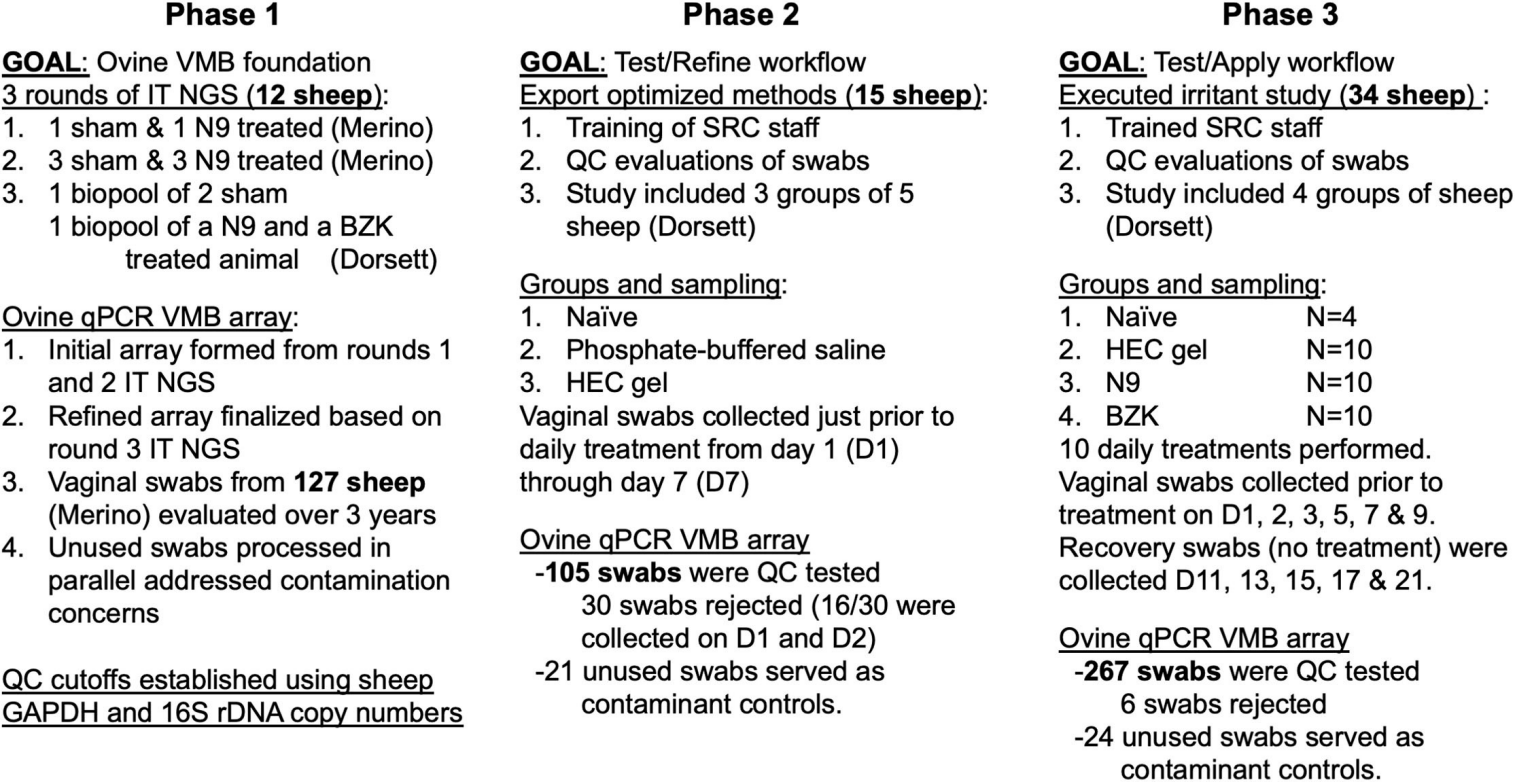
Overall study design for the three phases of the study. The iterative approach, completed over 8 years, first established collection and analysis methods (phase 1) in two breeds of sheep housed under different conditions. The optimal methods were then exported to a second research team at a second site (phase 2), allowing for refinement of techniques and confirmation of sample shipment methods before completing an expanded evaluation (phase 3) of irritants selected to impact the ovine vaginal environment with the expectation of shifted vaginal microbiome (VMB) and inflammation development to identify the most common microbiota changes and their association with inflammatory markers.

A three-phase study design was employed to ensure optimal quality of all the samples and a broad dataset from which a conclusion was drawn. The phase 1 studies at UTMB completed on Merino sheep housed in environmentally controlled indoor housing produced basic methods for sample collection and handling prior to the initiation of a phase 2 pilot study at SRC on Dorset sheep. Three groups of vaginal swabs from a total of eight Merino and four Dorset sheep were collected as either sham, N9- or BZK-treated representatives and analyzed by Ion Torrent 16S NGS (below) returning sequence for 95% of the bacterial 16S rDNA (35, 36). These data as well as initial analyses of bacteria detection using human VMB PCR targets (4, 5) led to the production of a customized ovine VMB qPCR array (below). Over 100 additional phase 1 Merino sheep samples and 10 mock swab samples were analyzed on the custom sheep VMB array to create the foundation datasets, quality cutoffs, and sampling methods. The phase 2 study on the Dorset animals directed refinements of the methods performed by staff at SRC as well as confirmation of molecular quality cutoffs. The phase 2 study included three groups of five animals (sham, phosphate-buffered saline, PBS, or HEC gel treatment) sampled daily for 7 days prior to treatments, producing a total of 105 vaginal swab samples tested on the sheep qPCR array. The final phase 3 study tested the impact of vaginal applicants (HEC placebo gel, 4% N9 gel (Conceptrol™), or 0.2% benzalkonium chloride (BZK) solution on the VMB after optimized methods were in place at SRC. In this study, the test compounds were administered intravaginally daily for 10 consecutive days (main period). Designated animals (four per group) were followed through a non-treated recovery period of an additional 11 days. The primary outcomes for this GLP study on the treated and naïve animals were vaginal health using the modified DRAIZE scoring system commonly used in rabbit vaginal irritation studies (ISO 10993-10). Periodically during the main and recovery periods (main: days 1, 2, 3, 5, 7, and 9; recovery: days 11, 13, 15, 17, and 21), vaginal swab samples (*n* = 264) were collected and processed (below) for VMB analysis as well as cytokine ELISA. This overall three-phase study design is shown in Figure 1.

### Vaginal Sampling

Sterile calcium alginate swabs (Thermo Fisher, Sugar Land, TX, United States) were removed from the pack and inserted into the vaginal cavity of each animal by a trained handler wearing gloves, taking care to avoid contact with any external site. The animals were shorn as needed to limit contamination by contact with wool. Swabs were collected each morning prior to administration of any test applicant and at least 20 h after the prior application. The inserted swab was passed over vaginal lateral walls approximately five times per side before removal and placement into 2 ml of sterile DPBS in sterile, pre-labeled 15 ml conical tubes. The tubes were stored on wet ice until taken to the lab for subsequent processing in a biosafety cabinet. Parallel mock swabs were collected and analyzed for each study phase. In a sterile cabinet, the closed 15 ml conical tube was vortexed for 10 s prior to the transfer of 100 ul of fluid to a cryotube filled with 100 ul of external lysis solution (Roche, Indianapolis, IN, United States). The remaining fluid and the cryotube were placed at −80C. For the phase 3 study, an additional aliquot was created for subsequent ELISA and frozen separately.

### VMB Characterization

Aliquoted lysates of vaginal fluids (200 ul) were thawed and then deposited into individual wells of 96 deep-well plates before the DNA was extracted in a high-throughput fashion using a MagNA Pure 96 instrument (FDA approved for IVD) in combination with the MagNA Pure 96 DNA and viral small volume-IVD isolation kit (Roche). Residual DNA was aliquoted and archived, and used for subsequent qPCR assays described below to refine the VMB CT profiles. The initial step in the sample evaluation assessed the quality of the recovered DNA by qPCR for the single copy sheep GAPDH (shGAPDH) and a “universal” bacterial 16S rDNA (16S) PCR target (30). This outcome was established if any sample was below established limits for inclusion. Quality data also provided an indication of the overall success of the study and the impact of vaginally applied articles on the total bacterial burden (universal 16S level) in the samples. These quality metrics were repeated after thawing to confirm sample integrity as part of the custom qPCR array to quantify any degradation during the −20°C storage. Each sample was required to be within 0.5 log10 of the original values to be included in subsequent analyses. In cases where samples failed established metrics, they were omitted from further consideration. For every analysis run, sensitivity and quality standards were included as controls to address technical concerns. This included every step in the pipeline from initial sample collection (lot validation of bacterial-genome free-swabs, mock swabs, and DPBS) to qPCR array (known positive and negative control samples are run on each production run of assembled arrays) and cytokine analyses (synthetic standards, experimental standards, and negative controls).

### VMB 16S NGS

The phase 1 VMB 16S NGS was carried out on eight Merino and four Dorset vaginal samples (Figure 1) using a fusion-PCR method and an Ion Torrent Personal Genome Machine (PGM) NGS platform. Briefly, fusion primers were designed in accordance with the guidelines of the manufacturer (Ion Amplification Library Preparation – Fusion Method, Life Technologies, Carlsbad, CA, United States) using Ion Xpress Barcodes linked to 16S gene primer pairs targeting hyper-variable regions 1–8 (37, 38). Each 25 μl PCR amplification contained: 12.5 μl iQsupermix™ (Bio-Rad, Hercules, CA, United States), 1 μl of each forward and reverse (5 μM) primer, 9.5 μl nuclease-free water, and 1 μl of DNA template. DNAs from a total of 12 disparate sheep vaginal swab samples were used as templates for the creation of 10 fusion 16S libraries. The eight Merino samples were processed individually and included four sham and four N9-treated animals. The four Dorset samples were processed as two biopools; one pool was two sham-treated sheep vaginal swabs. The second pool was one N9- and one BZK-treated animal. Fusion PCR was completed in a c1000 thermocycler (Bio-Rad) using the following parameters: Cycle 1), 95°C, 3 min, Cycle 2), Step 1. 95°C, 45 s, Step 2. Primer-specific annealing temperatures, 45 s, Step 3. 72°C, 2 min repeat 39 ×, Step 4. 72°C for 7 min. PCR products were purified using Qiagen Qiaquick spin columns and quantified using a spectrophotometer (Bio-Rad). The PCR products were then diluted, mixed in equal proportion, and sequenced on an Ion Torrent PGM using 400 base pair read kits together with 316 size chips following the instructions of the manufacturer (Life Technologies).

After generation, sequencing reads were filtered for quality and binned according to Ion Xpress barcode using Ion Torrent Suite software version 5.0.5. Sequencing reads in the FASTQ format were further processed using the web-based Galaxy software (39). First, raw FASTQ files were normalized using the FASTQ groomer tool function. Next, each barcoded read was trimmed to remove the primer sequence and subsequently filtered to the expected size of the 16S gene target. After this level of processing, the sequence reads were concurrently compared to the SILVA 16s database using the bowtie 2 software (40, 41). This yielded a call to genera level as well as the number of times each sequence matched the database. Where multiple calls to the same genera were made, the number of hits was summed accordingly. These numbers were converted to the percentage of the total to give an overall ratio of individual bacterial types in the sequenced VMB. FASTQ data files are available at NCBI SRA (bioproject accession number PRJNA733201).

### Creation of a Custom QPCR Sheep VMB Array (VMBA)

Based on the NGS data, a core VMB was determined identifying the most common genera and species of bacteria present in the 12 vaginal samples in phase 1. These bacteria represented > 90% of the total bacteria identified in the VMB. From the core VMB, a customized qPCR array was developed to evaluate 46 VMB community member targets using our previously published method (42). Two additional control targets were included to confirm the quality of the DNA at the time of testing and to confirm the proper loading of the VMBA. Specifically, primer pairs that amplified shGAPDH and universal 16S rDNA were included to create a total of 48 targets analyzed for each sample. The array was constructed in a 96-well plate (Thermo Fisher Scientific Inc.) in a 6 × 8 format, allowing for the evaluation of two samples per 96-well plate. Each 25 μl PCR was carried out using 12.5 μl iQ SYBR Green Supermix™ (Bio-Rad), 1 μl of each forward and reverse (5 μM) primer, 9.5 μl nuclease-free water, and 1 μl of DNA template. qPCR was completed in a c1000 thermocycler equipped with a CFX™ reaction module (Bio-Rad) using the following parameters: Cycle 1), 95°C, 3 min, Cycle 2), Step 1. 95°C, 30 s, Step 2. annealing/extension 60°C, 1 min, repeat 39 ×, Step 3. 72°C for 2 min, Step 4. Melt-curve 74–90C, 0.2°C temperature increments with 5 s plate read the time. Fluorescent signal data were collected at the end of each annealing/extension step. Starting quantity values were extrapolated from standard curves of plasmids harboring the qPCR targets. Mathematical analyses were performed using Excel™ (Microsoft Corp., Redmond, WA, United States) with a customized macro template file developed to complement the array. The qPCR data were converted to genome counts through linear regression equations derived from sequence-confirmed plasmid clones of each qPCR target. During phase 1, the qPCR array was used to analyze 127 vaginal swabs collected over 3 years. The quantified 16S rDNA copy number indicated the total bacterial load. The sum of the specific bacterial targets was compared to the 16S copy showing that the finalized qPCR array accounted for >90% of the 16S copy for all of the samples serving as an internal measure of organisms not targeted by the VMBA.

To convey VMB profiles and establish community types (CTs), experimental sample copy numbers were converted to the percentage of total bacterial genomes to create proportional bar charts to visually convey the individual sheep sample VMB profile. Normalized genomic counts were also compared using a non-centered clustering algorithm (43). This clustering analysis was also used to identify biomarker, dominant bacteria community members. For the establishment of natural CT categories, all the baseline, pre-dose (PD), and the Sham group vaginal samples from Merino and Dorset ewes were combined into an analytical set designated as “No Tx (PD + Sham)” (*N* = 71 sheep).

For the study on the impact of vaginal applicants on VMB, the established CTs were then used to compare between treatments (Sham and HEC Placebo groups generally served as control comparators but all possible comparisons were performed among the four groups). After the generation of the data, there were additional opportunities for stratification and categorization based on VMB shifts related to the treatments. Furthermore, *via* additional clustering and paired *t*-test analyses, two novel CTs were identified based on the grouping of similarly treated animals. Furthermore, the identified VMB CT biomarker genera or species have become datasets within the larger groups, allowing for comparisons of absolute quantities. If associated with inflammation states, such differences could be predictive of susceptibility to infection or impactful of vaginal applicant efficacy.

### Sheep Cytokine ELISAs

Ovine vaginal samples were evaluated for IL-1β, IL-6, IL-8, IL-17α, CXCL10, and TNF-α cytokine levels using previously published methods (28) with slight modification to facilitate high-throughput processing using a TECAN Freedom Evo 8 tip liquid handling robot outfitted with a ROMA arm. Our automation of these custom ELISAs made use of commercial standards, as well as synthetic and experimental positive and negative controls. Validation of the conversion from manual to automated processing for each ELISA was completed using synthetic standard and produced indistinguishable standard curves other than improved precision with the automated processing (data not shown). The scripting coordinated the use of an onboard Hydrospeed automated plate washer and integrated a Magellan M1000 plate reader. Initial aliquoting of the remaining ovine vaginal fluid in 15-ml conical tubes into eight daughter plates in a 96-well format (120 ul/well) was also completed using the TECAN system. Plates 1–6 were utilized for the six target-specific ELISAs without the need for successive rounds of thawing, increasing the quality of the data. Preliminary qualification and comparison experiments for each assay using synthetic standards produced indistinguishable results from manual or automated ELISA processing, confirming the utility of the automated approach. For the final assays, synthetic standards were employed with a range of 0.034–25 ng/ml to test the robustness and reproducibility of each of the ELISAs. Each plate processed included a series of diluted standards to ensure quality and reproducibility across the sample set.

Briefly, for the ELISA, Immulon 2 HB flat-bottomed 96-well plates were coated with the appropriate cytokine capture antibody at a concentration of 5 μg/ml, 50 μl per well, before being sealed and incubated overnight at 4°C. Peptide standards were serially 3-fold diluted to produce a dynamic range consistent with expected levels of cytokines in vaginal fluids (28). Negative reagent blanks were used to address background and specificity in each assay. The positive and negative controls were placed in duplicate columns on each plate by TECAN liquid handling using conductive liquid sensing disposable filter tips. Experimental samples from daughter plates (a volume of 100 μl) were then transferred by the TECAN system into predetermined wells. Details of the antibody source and dilutions are provided as Supplemental Information or in the prior publication (28). The IL-6 and IL-8 ELISA used unconjugated detection antibodies. The IL-1β, IL-17α, CXCL10, and TNF-α ELISAs utilized biotinylated detection antibodies. For the IL-6 and IL-8 ELISA, 100 μl of an HRP-conjugated secondary antibody (rabbit IgG; Abcam, Cambridge, United Kingdom) was used. For the ELISAs that utilized biotinylated antibodies, 100 μl of streptavidin-peroxidase (Sigma-Aldrich, St Louis, MO, United States), diluted 1:2,000, was employed. Plates were developed with a solution containing 5 mg o-phenylenediamine dihydrochloride (Sigma-Aldrich) dissolved in 10 ml of citrate buffer (pH 4.2) containing 10 μl of 30% hydrogen peroxide. The reaction was halted with the addition of 50 μl of 1N sulfuric acid. Colorometric data were subsequently acquired through the reading of each plate at a wavelength of 490λ on a Cytation 3 (Biotek, Winooski, VT, United States) plate reader.

This panel of inflammatory markers was chosen because they have been reported in preclinical studies to indicate signs of vaginal mucosal inflammation and toxicity (1, 3, 7, 44) and to be associated with increased risk of HIV acquisition in women (45). Kinetic vaginal sampling in the phase 3 study produced a dataset (*n* = 267 vaginal swab fluids) of natural and induced VMB dysbiosis and eubiosis. In addition, 30 previously characterized sheep vaginal samples from unrelated studies were included as plus/minus quality metrics because these samples had been subjected to repeated freeze/thaw. For each cytokine target, a total of four plates were processed, and each contained 80 experimental samples and 16 controls. Two plates were loaded with a high range of standards (seven dilutions) and a buffer-negative control in duplicate using 16 wells. The second two plates were loaded with a low range and negative control in a similar fashion, providing a total range of 0.034–25 ng/ml for each synthetic standard and nine distinct concentrations to plot standard curves (data not shown).

### Statistics and Data Analyses

Quality data (shGAPDH) were log10 transformed, and the log fold change to baseline (Day 1; D1) was calculated per animal. Daily and maximum (main and recovery Periods) log fold change in shGAPDH levels were summarized, in general, using descriptive statistics including the number of observations (n), mean, SD, median, interquartile range, minimum, and maximum. Daily and maximum (phase 3, main and recovery periods) log fold change in shGAPDH levels were tested for normality (e.g., Shapiro–Wilk test). Daily and maximum (main and recovery periods) log fold change in shGAPDH levels were compared for treatment effects by either parametric (e.g., ANOVA) or non-parametric (e.g., Wilcoxon) methods. Pairwise comparisons between groups were adjusted (Tukey's or Dunnett's test) for multiple comparisons.

To establish the impact of 16S levels across the HEC, N9, BZK, and Sham treatment groups, the data were again log transformed, and the log fold change to baseline (D1) was calculated per animal. Daily and maximum (main and recovery periods) log fold change in 16S levels were summarized, in general, using descriptive statistics including the number of observations (n), mean, SD, median, interquartile range, minimum, and maximum. Daily and maximum (main and recovery periods) log fold change in 16S levels were tested for normality (e.g., Shapiro–Wilk test). Comparison of the group 16S outcomes again evaluated daily and maximum (main and recovery periods) log fold change in 16S levels to identify treatment effects by either parametric (e.g., ANOVA) or non-parametric (e.g., Wilcoxon) methods. Pairwise comparisons between groups were adjusted (Tukey's or Dunnett's test) for multiple comparisons.

VMB profiles for each sample from each of the four animal groups that met quality criteria were assigned a CT and statistically compared (standard paired *t*-test comparisons) to their individual animal baseline VMB profiles to identify the bacteria that were impacted by the treatments. The rationale for this approach is like that used in clinical research in which samples were obtained from a variety of women, and then classified into community state types (13). Bacterial CT frequency data were summarized, in general, using percentages for main and recovery periods. Bacterial CT frequency distributions were compared among the HEC, N9, BZK, and Sham treatment groups by asymptotic, two-sample, Kolmogorov–Smirnov test (46–48). This provided a *p* value for each comparison/period. The *p* value was adjusted for multiple comparisons during main and recovery periods. In addition, Fisher exact tests were performed between pairs of treatment for each community type, treatment, and period. The Benjamini–Hochberg correction for genomic data (49) was used to determine the significance of the Fisher exact comparisons. Finally, the relative abundance (% RA) of selected organisms (PCR targets) were compared across treatment groups to identify any organism specifically impacted by the applicant. The Shannon Diversity Index (SDI) was calculated for each sample for each group and main and recovery study periods. The % RA was compared among the HEC, N9, BZK, and Sham treatment groups, per time point, by non-parametric Wilcoxon tests. Pairwise comparisons between groups were adjusted (Tukey's or Dunnett's test) for multiple comparisons. An additional “two statistics” approach (50) was used to avoid reductions in the power of the Wilcoxon test due to tied ranks of 0% values. The two-statistic approach included a *Z* test to compare the proportion of zeros followed by a Wilcoxon comparison on the non-zero values that produced a *p* value with two degrees of freedom. SDI was compared between groups by Welch *t*-test to allow for unequal variances.

## Results

### Study Design

Using a three-phase approach (Figure 1), we determined the profile of the ovine vaginal microbiome (VMB) in the Merino sheep studied in an environmentally controlled indoor housing facility and the Dorset sheep maintained in open-air housing. The animals were provided unlimited access to food and water but received different diets that, for the Dorset sheep, included hay in contact with the floor. The Merino sheep received a mixture of pelleted food and hay that was removed from the caging *via* slotted flooring and automated cage washing. Initial phase 1 studies were completed on vaginal swabs from naive Merino animals housed throughout the year at UTMB over a 3-year time frame (Figure 1). The second phase of the project exported optimized methods and sample collection techniques to the SRC site, allowing us to refine training and improve sample success before initiating the final phase of our study that included vaginal application of three test articles expected to alter the VMB and impact vaginal health (Figure 1). Through the final phase 3 study that included applicant-based induced inflammation and custom ELISAs, we identified VMB community types (CTs) that were associated with dysbiosis and others that were linked to eubiosis and vaginal health. The selected applicants were: (1) HEC placebo gel, designed as a minimally perturbing control formulation and is commonly used as the “universal placebo” in microbicide safety studies based on its physical properties, stability, and *in vitro* and *in vivo* biological activities; (2) N9 (Conceptrol™) 4%, a common contraceptive agent presents in most spermicide products such as vaginal gels, creams, foams, suppositories, sponges, and films. Although N9 is considered safe for use as a contraceptive agent, it has been shown to cause reversible disruption of the vaginal epithelium and is considered a reference standard for vaginal safety studies; (3) BZK 2%, which also has a spermicidal capacity similar to N9 and is sold in contraceptive suppositories and creams. BZK is well-known to cause tissue injury at higher concentrations in women in clinical studies; therefore, it is considered a known irritant for vaginal safety studies at concentrations ranging from 0.2 to 2% that have been shown to cause disruption of the mucosal epithelial barrier and inflammation in sheep (21, 22, 28).

### Establishment of Metrics to Monitor Quality and Consistency of Vaginal Sampling Across two Sites and two Breeds of Sheep

As part of this project, historical quality metrics were applied to the data, confirming the established exclusion criteria for the quality of vaginal swabs. Specifically, extracted DNA must include at least 1 × 10^4^ copies/ul of the single copy ovine gene, shGAPDH, and 1 × 10^4^ copies/ul of the “universal” bacterial target (16S rDNA) *via* qPCR for the sample to be included in subsequent analyses. For the initial work with the Merino sheep samples, all 127 surpassed the inclusion metrics. Using this set of cutoffs, the assessment of swab collection was possible, as evidenced by the completion of the phase 2 pilot study at SRC. After communication of the methods, 105 swabs were collected from 15 Dorset sheep over a 7-day sampling period. After quality assessment, 30 (22%) swabs were excluded from any subsequent analyses. Half of the excluded samples were collected during the first 2 days of the study, suggesting that groups developing the model should be encouraged to complete initial testing with the system and an ongoing need for training of all animal handlers. Consistent with the experience gained from the pilot study, the quality of vaginal swab samples greatly improved for the phase 3 study, where only 2.2% (6/267) of the collected swab samples failed to meet the minimum quality criteria and were excluded from further analysis.

**Figure 2:**
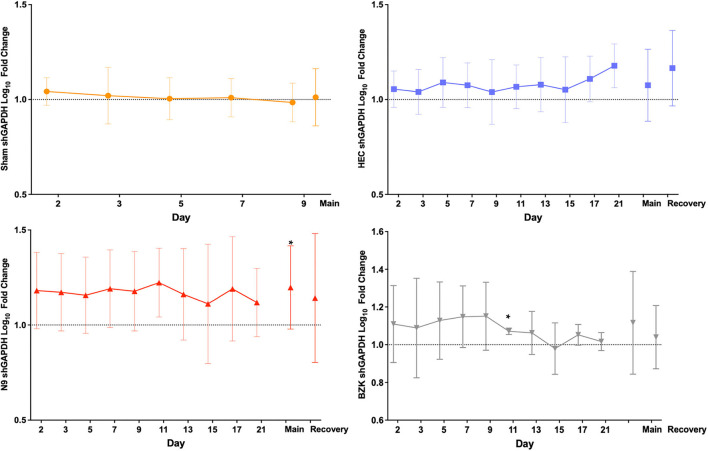
Average log_10_ fold change from baseline levels of sheep vaginal cell counts (shGAPDH). The impact of Sham (top left), hydroxyethylcellulose (HEC) placebo gel (top right), nonoxynol-9 (N9) gel (lower left), or benzalkonium chloride (BZK) solution (lower right) applied daily for 10 days to the sheep vaginal cavity was assessed by collection of vaginal swabs during phase 3 of the study. Each swab was processed for shGAPDH loads by quantitative polymerase chain reaction (qPCR) that were log_10_transformed and compared to the individual day 1 (D1) pre-dose baseline for each animal. The fold change was then averaged by the group for all study days in both the treatment (main, D1–D10) and recovery (D11–D21) periods of the study, and plotted with standard deviations. The maximal difference from baseline for the main and recovery periods are shown as individual values on the right side of each graph for the three treatment groups where recovery period samples were collected. Daily and maximum (main and recovery periods) log_10_ fold change in shGAPDH levels were tested for normality (e.g., Shapiro–Wilk test). Daily and maximum (main and recovery periods) log_10_ fold change in shGAPDH levels were compared for treatment effects by either parametric (e.g., ANOVA) or non-parametric (e.g., Wilcoxon) methods. Pairwise comparisons between groups were adjusted (Tukey or Dunnett) for multiple comparisons. * = *p* < 0.05. Non-transformed average daily values are presented in Supplementary Figure 1.

Analysis of the shGAPDH levels of the 261 included vaginal swabs from the final study provided the opportunity for several evaluations of the impact of the treatments. Quantified shGAPDHs were studied as indirect indicators of the molecular health of the vaginal mucosa over the main and recovery study periods but primarily indicated sample quality for our studies. Sheep cells collected by vaginal swabs can indicate either shedding of damaged layers of tissue or active repair of the mucosa damaged by the daily application of the treatments. As shown in Figure 2, the log_10_ fold change from the baseline (day 1, D1) pre-dose (PD) sample for each individual animal was calculated for each day and then averaged across the animals in a treatment group and plotted over time. The results illustrated no significant between-group changes with a trend toward slight elevation in those animals that received any of the applicants (HEC, N9, or BZK) relative to the Sham controls. The greatest trend was observed in the N9-treated animals, but this was not significantly different from the other groups. These comparisons did not support conclusions of any obvious changes in the mucosal health of the treated animals but suggested that more cells were shed and captured by the swab in animals that received an irritant article material. Comparison of the maximal log_10_ fold change data for each group during the phase 3 main and recovery periods did not reveal statistical differences from baseline for any group other than the N9 treatment group (Figure 2). In the N9 dataset, a significant increase (*p* < 0.05) was observed in the maximal shGAPDH level during the main period consistent with the daily elevations seen that individually were not found to be significantly different from baseline as noted above. An important outcome of this analysis was the indication that vaginal swabbing in the Sham-treated animals did not lead to any significant changes in the vaginal mucosa in this study (Figure 2). This supports the conclusion that the frequency of vaginal swab collection did not have a measurable impact on the health of the mucosa.

**Figure 3:**
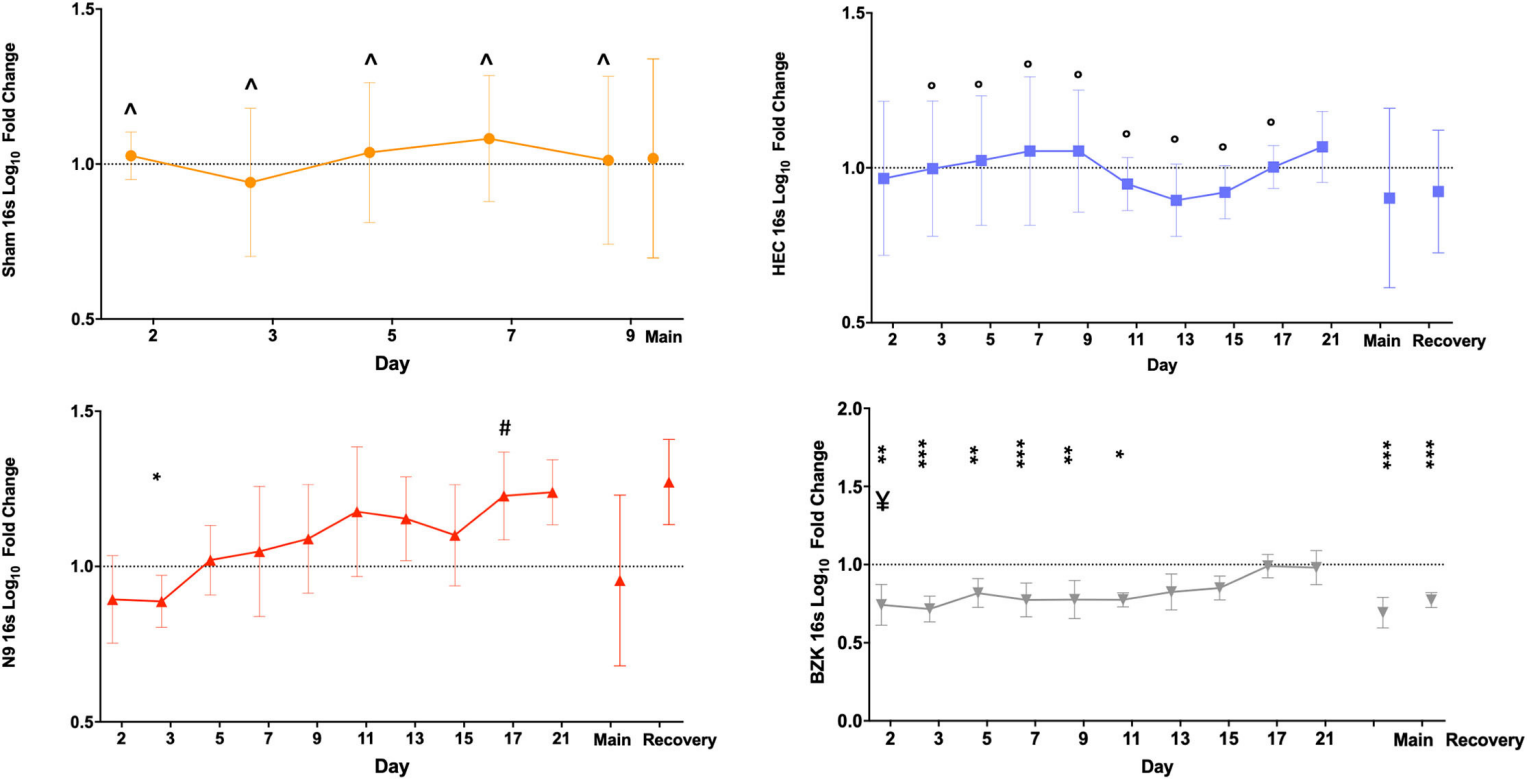
Average log_10_ fold change from baseline levels of vaginal bacteria genome counts(16S). Similar analyses were completed on the same swab samples to determine the impact of Sham (top left), HEC placebo gel (top right), N9 gel (lower left), or BZK solution (lower right) applied as described for shGAPDH evaluations. Each plotted point is the averaged fold change with standard deviations. The maximal difference from baseline for the main and recovery periods are again shown as individual values on the right side of each graph for the three treatment groups where recovery period samples were collected. Comparisons were as described for the shGAPDH analyses in Figure 2. Statistical differences from group baselines are shown as * = *p* < 0.05; ** = *p* < 0.01; *** = *p* < 0.0001 and are more fully described in the results. Pairwise differences between groups were adjusted (Tukey's or Dunnett's test) for multiple comparisons, and *p* < 0.05 findings are indicated with the following comparisons showing statistically significant differences: BZK vs. Sham for D2, 3, 5, 7, and 9; BZK vs. HEC for D5, 7, 9, 11, 13, 15, and 17; BZK vs. N9 for D2; N9 vs. HEC for D17. Non-transformed average daily values are presented in Supplementary Figure 1.

Similarly, the 16S rDNA level quality metric provided an indication of the impact of vaginally applied irritants or toxicants on the microbiota. As described for shGAPDH data, the log_10_ fold change from the D1 baseline PD sample for each animal was calculated for each day and then averaged across the animals in a treatment group and plotted over time (Figure 3). The results indicated that neither repeated vaginal swab collection (Sham) nor repeated sampling in the context of daily dosing of the placebo HEC gel caused measurable changes in total bacterial loads (Figure 3), further supporting the sampling methodology. As predicted by prior results, the BZK irritant significantly impacted the total bacterial load. In fact, after only a single BZK application, significantly lower average bacterial genome loads (D2) compared to either the Sham or HEC-treated groups (*p* < 0.01) were observed. Bacterial burden was significantly decreased on D2, 3, 5, 7, and 9 compared to the HEC Group (*p* < 0.05). BZK treatment results were also significantly different on D5, 7, 9, 11, 13, 15, and 17 compared to the N9 group (*p* < 0.05), supporting a more substantial impact of this irritant. The N9 treatment led to 16Slog_10_ fold change relative to the animals in the HEC group only on D17 (*p* < 0.05). Interestingly, the N9 treatment did not produce significantly different bacterial burdens compared to baseline or to the control groups but trended higher over the course of the study, suggesting a level of overgrowth of total bacteria during the main and recovery periods. In comparison to BZK, the N9 treatment produced significantly higher levels of bacteria compared to the group baseline at nearly every time point tested (Figure 3).

As shown in Figure 3, after cessation of treatment, the BZK-treated animals slowly returned to original bacterial genome levels, reaching baseline values by D17 (7 days after the last vaginal application). In contrast, the N9 animals trended higher than baseline levels, but these differences were not significant. Also noteworthy were the results for the maximal log_10_ fold change during the main and recovery periods. Consistent with daily comparisons, the BZK-treated animals showed significantly lower levels of total bacterial content (*p* < 0.05) for both time periods when maximal log_10_ fold change was calculated for the group (Figure 3). The non-transformed daily averages for both shGAPDH and 16S levels for each group are provided in Supplementary Figure 1.

### VMB Profiling, Identification of Marker Organisms, and Assignment of Community Type (CT) Categories

**Figure 4:**
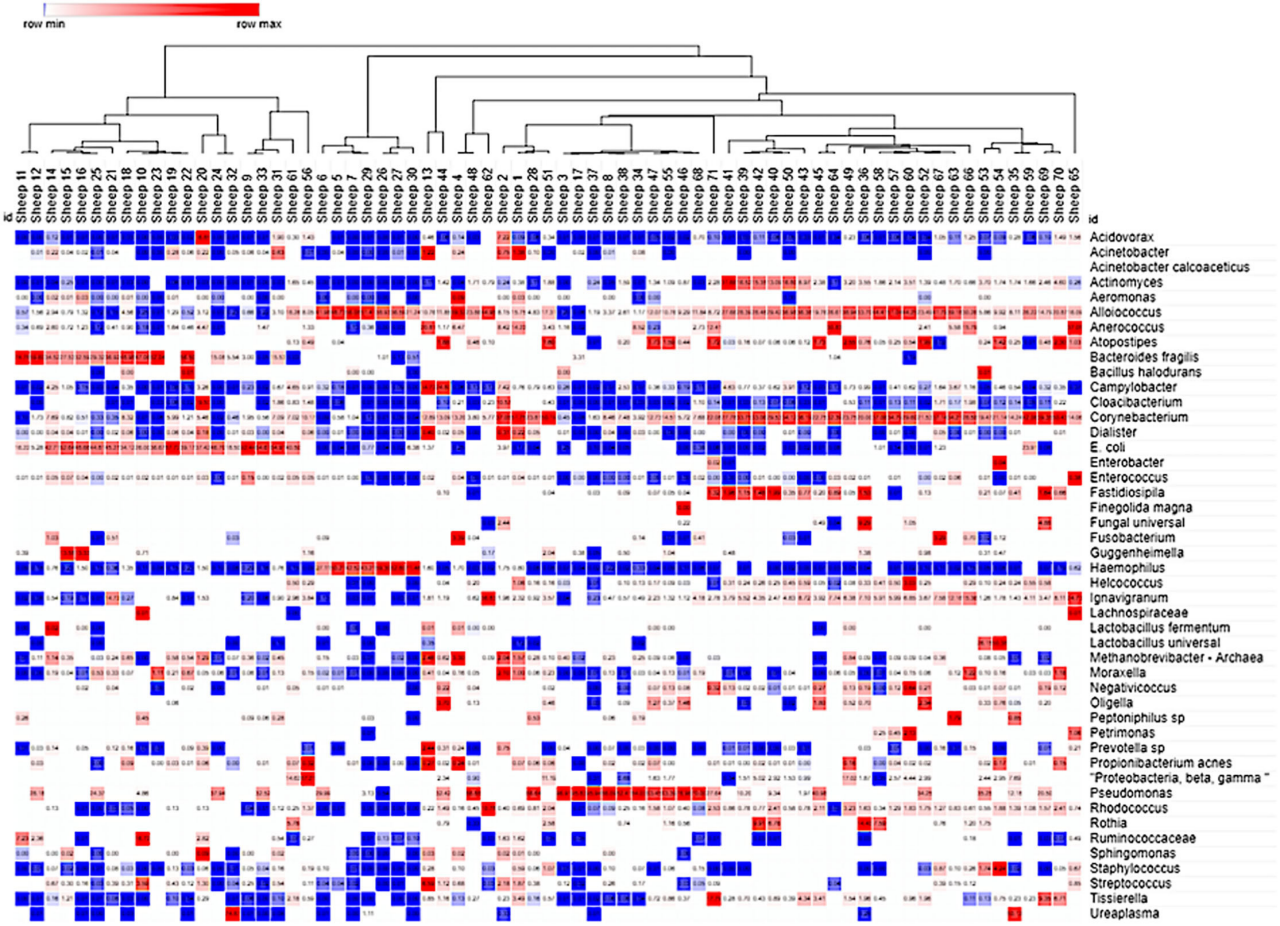
Cluster analysis of the ovine VMB in naïve and Sham-treated animal samples. Heat map clustering [unsupervised using Morpheus online (43)] illustrated dominance by individual or combinations of bacteria as well as grouping of samples into community type (CT) categories indicated by branching shown across the top of the Figure. Proportional levels of the bacteria (right vertical labels) quantified by the VMBA are shown in rows with each sample (*n* = 71 distinct sheep) shown as a distinct column. Red boxes represent the maximal levels detected in a row, and blue shows the lowest detection levels. Clustering helped to identify CTs that were dominated by biomarker organisms that were confirmed by statistical comparisons, leading to the categorical CT system shown in Figure 5.

**Figure 5:**
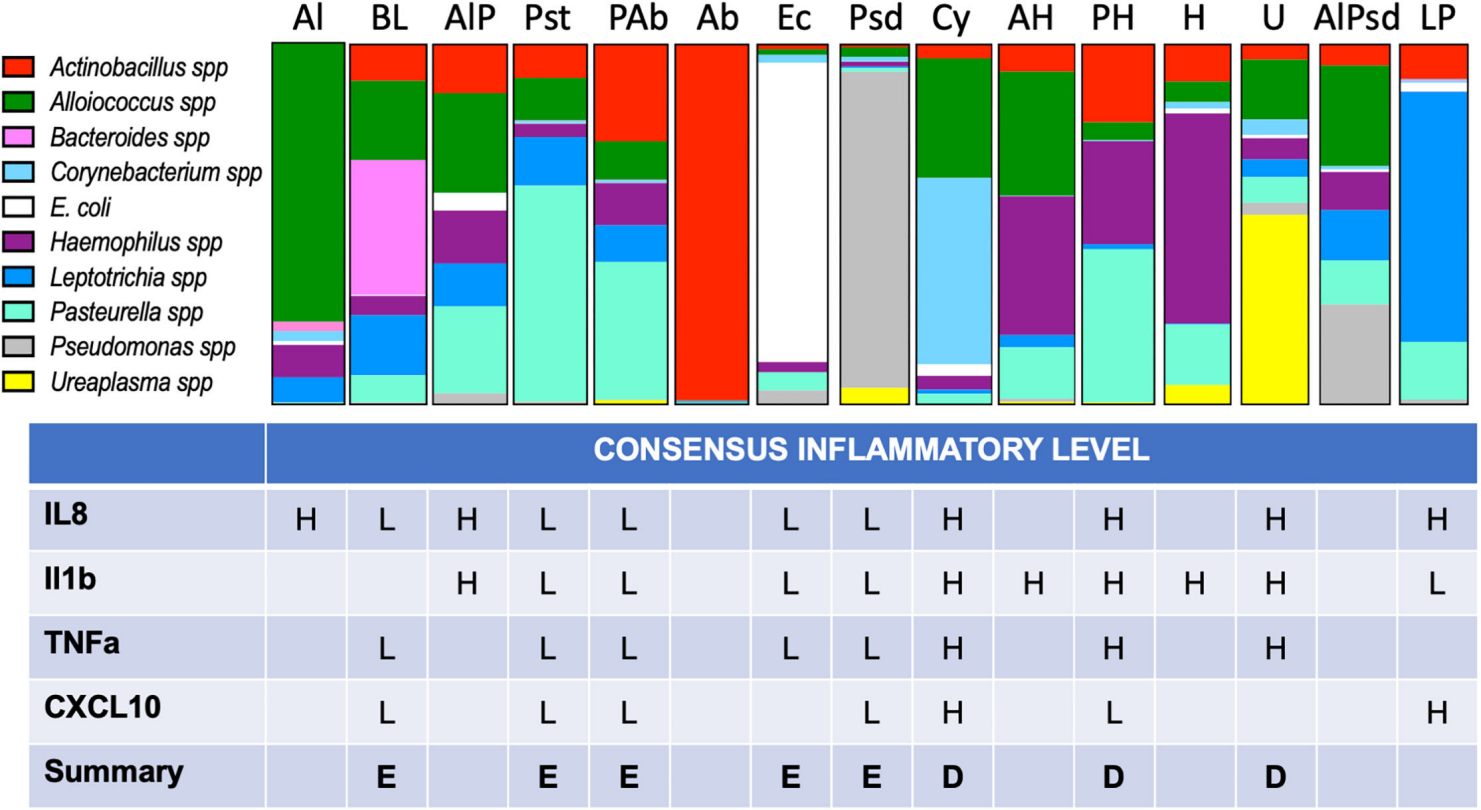
Proportional bar charts represent the average VMB community structure for each of the identified CTs and their inflammatory association. Top: The 10 identified biomarker organisms were proportionally charted to illustrate the average profiles from the 216 vaginal swab samples evaluated from phase 3 of the study. The number of representatives is shown below each bar. Bottom: Using the assigned CT, each of the individual swab samples was grouped (one sample was not typable by this system) and designated by one of several models of inflammation as described in the results. Using enzyme-linked immunosorbent assay (ELISA) data (Figure 6), we considered binary and tertile categorization schemes derived from all the samples regardless of CT for the four commonly detected targets. Using the binary distinctions of high (H) or low (L), assignments were made when at least 67% of the samples fell in one category. A final call of eubiotic (E) or dysbiotic (D) was made when at least three of the four targets showed consistency.

The molecular characterization of the VMB profiles was initially completed by NGS analysis of nearly the entire 16S rDNA sequence on an Ion Torrent PGM platformof bio-pooled swab samples from virginal (non-pregnant) female Merino and Dorset crossbred sheep (12 in total) that were either asymptomatic or showing signs of vaginal irritation naturally or through experimental methods. The collected data were used to create a custom qPCR array designed to quantify 90% of the core VMB microbiota in each sample through 46 distinct gene- or species-specific PCR targets arrayed into one half of a 96-well plate supporting the analysis of two samples per PCR run (VMBA). Vaginal swabs from 71 distinct “naïve” sheep were analyzed with the VMBA to identify marker genera or species to create a CT categorization system that was then applied to the experimental VMB samples. The molecular VMB profiles from baseline (pre-dose) and Sham-treated samples (No Tx PD + Sham) were subjected to mathematical clustering analyses to identify common (average) community types (CTs; Figure 4). After comparing the 16S value from the VMBA to the sum of all detected bacteria in some of the irritant-treated vaginal samples from the phase 2 and 3 studies, we determined that less than 90% of the microbiota were accounted for with the VMBA. This led us to complete additional Ion Torrent NGS on selected samples ultimately adding replacement targets to the final VMBA providing data that support a total of 16 CT categories (Figure 5).

Applying the CT categories to individual vaginal swab VMBA results collected in the phase 3 study, we categorized dominance shifts in ovine VMB in response to treatment with the irritants (N9 and BZK). During active treatment (main period), the CT distribution indicated that irritants N9 and BZK led to significant shifts in CT distributions when compared to the no treatment “No Tx PD + Sh combined group (*p* = 0.0006; Table 1). The BZK treatment also led to a significantly different distribution of CTs relative to the HEC treatment group (*p* < 0.001; Table 1). Consistent with the more substantial impact of BZK, there was a significantly different alteration in CT pattern compared to the N9 treatment (*p* < 0.05). No significant differences were found comparing CT distributions between the HEC placebo treatment and the No Tx combined group. This analysis also confirmed the identification of three CTs that were only observed in samples from the animals that received the irritant treatments (Table 1). Overall, these findings supported the use of CT categories as a simplified means of predicting VMB impacts by vaginally applied compounds in the ovine model during the active, daily application of the two selected test article irritants. These findings also supported the use of BZK as a control irritant article in future studies.

**Table 1:** Phase 3 vaginal microbiome community type (VMB CT) distribution, D1–D9 (main period).

**Group**	**Sheep VMB CT**						
	**A-H**	**Ps**	**Ec**	**H**	**Cy**	**U**	**Total**
Pre-Dose (PD)	26 (70.3)	6 (16.2)	5 (13.5)				37
Sham (Sh)	11 (78.6)	3 (21.4)					14
							
No Tx (PD+Sh)	37 (72.5)	9 (17.6)	5 (9.8)				*51
HEC	41 (75.9)	11 (20.4)	2 (3.7)				54
N9	30 (55.5)	1 (1.9)		17 (31.4)	5 (9.2)	1 (1.9)	54
BZK	20 (37.0)	9 (16.7)	2 (3.7)		20 (37.0)	3 (5.6)	54
Phase 3 Treatment Period Total (%)	128 (60.1)	30 (14.1)	9 (4.2)	17 (8.0)	25 (11.7)	4 (1.9)	213

Individual swab samples from the indicated groups were assigned to the indicated CTs and were statistically compared via Bonferroni–Holm adjusted by asymptotic, two-sample Kolmogorov–Smirnov test. Specific “p” values are reported in the text. Each cell shows the counts by group, with the percent of the swabs in the indicated CT shown parenthetically. ^*^The first two rows indicated no difference in the CT distribution of PD and Sh groups, allowing them to be combined as the “No Tx” group (N = 51) used for comparisons to the CT distribution during active treatment (main period D1–D9) for each of the three treatment groups.

CT distributions were also studied after treatments were completed (phase 3 recovery period). The HEC treatment did not create a significant difference between the main and recovery period CT distributions (Table 2; *p* > 0.05, Benjamini Hochberg adjusted Fisher exact test) with the noted presence of 2/16 samples in what appeared to be a transitional CT (A-H-Ps). As noted above, the lack of impact of the HEC placebo during the main period did not “recover” to a normal state. In contrast, the N9 treatment led to shifted CT distribution that, during the recovery phase, returned to a state more like the HEC or No Tx combined group data. As a result, a significant shift (*p* < 0.05) in CT distribution was observed consistent with a lack of sustained impact and a return to a pre-treatment distribution (Table 2). Finally, evaluation of the CT distribution in BZK main and recovery samples was found to be statistically similar, supporting a sustained impact of the BZK during the period from D11 to D21 (*p* > 0.05). Using the CT results, biomarker organisms were associated with treatment outcomes. Specific comparisons of CT distribution revealed that there was a higher frequency of the A-H CT for the No Tx (PD + Sh) group compared to the BZK Group (Table 1 vs. Table 2; *p* < 0.01). A higher frequency of the A-H CT was found during the main vs. recovery period for the N9 and HEC groups (*p* < 0.05). A higher frequency of the Ps CT was found for the No Tx combined group compared to the N9 group (*p* < 0.05). The H CT was found more frequently for the N9 group compared to both the No Tx and BZK groups (*p* < 0.0001). Interestingly, the H CT was found for the N9 Group during the main period only making a significant difference with the respective recovery data set (*p* < 0.0001). The BZK treatment was associated with a higher frequency of the Cy CT compared to both the N9 and No Tx (PD + Sh) groups (*p* < 0.01). There were no significant differences in Ec and U CT frequency in either the main or recovery phases (*p* > 0.05).

**Table 2:** Phase 3 (main vs. recovery period) VMB CT distribution.

**Group**	**Sheep VMB CT**							
	**A-H**	**Ps**	**Ec**	**H**	**Cy**	**U**	**A-H-Ps**	**Total**
HEC Main	41 (75.9)	11 (20.4)	2 (3.7)					54
Recovery	6 (37.5)	8 (50.0)					2 (12.5)	16
HEC Total	47 (67.1)	19 (27.1)	2 (2.9)				2 (2.9)	70

N9 Main	30 (55.5)	1 (1.9)		17 (31.4)	5 (9.2)	1 (1.9)		54
Recovery	16 (100)							16
N9 Total	46 (65.7)	1 (1.4)		17 (24.3)	5 (7.1)	1 (1.4)		70

BZK Main	20 (37.0)	9 (16.7)	2 (3.7)		20 (37.0)	3 (5.6)		54
Recovery	9 (56.3)	1 (6.3)	1 (6.3)		2 (12.5)	1 (6.3)	2 (12.5)	16
BZK Total	29 (41.4)	10 (14.3)	3 (4.3)		22 (31.4)	4 (5.7)	2 (2.9)	70

*As described in Table 1, individual swab samples were assigned to indicated CTs and then statistically compared via Bonferroni–Holm adjusted by asymptotic, two-sample Kolmogorov–Smirnov test. Specific “p” values are reported in the text. Each cell shows the counts with the percent of the total shown parenthetically. The CT distribution for both of the phase 3 study periods is shown to illustrate any durable impacts of the applicants. The findings for N9 main vs. recovery were significantly different with p = 0.0458, but the other main vs. recovery comparisons were not significant*.

In addition to the CT analyses, because the VMBA provided quantitative outcomes, relative abundance (% RA) comparisons were performed for each of the 46 targets that were proportionally considered in the context of the total detected bacteria in the VMBA. The % RA created summary statistics that were addressed through both Wilcoxon and two test comparisons. These comparisons that revealed relevant, significant changes have been summarized to aid the understanding of the utility of the VMB outcome measure in the ovine vaginal irritation model. The animals were identified with disparate VMBs at the PD D1 time point that would have impacted subsequent evaluations of the impact of the treatments or sampling. Evaluation of the % RA by both Wilcoxon and two test methods revealed no incidences of significant group effects on PD D1. Significant effects (*p* < 0.05) found by the Wilcoxon and Two Test methods were identified on D2, 3, 5, 7 9 during the Main Period supporting direct causation of the test articles on selected bacterial targets. These specific % RA changes are provided in a summary table (Table 3).

**Table 3:** Summary of organisms that were observed to have significantly increased relative abundances (%RA) between phase 3 study groups.

**Group comparison**	**Study day**				
	**2**	**3**	**5**	**7**	**9**
N9 v HEC	*>Actinomyces*			*>Haemophilus* *>Helcococcus*	
BZK v HEC		*>Acidovorax* *>Corynebacterium*		*>Corynebacterium* *>Helcococcus* *>Rhodococcus*	*>Cloacibacterium >Corynebacterium >Helcococcus >Rhodococcus*
N9 v Sham				*>Helcococcus*	
BZK v Sham				*>Helcococcus*	
Sham v N9			*>E. coli*		
HEC v N9				*>E. coli*	
N9 v BZK				*>Haemophilus*	*>Haemophilus*
BZK v N9					*>Cloacibacterium >Corynebacterium >Rhodococcus*

*Following the assignment of CTs to eubiotic or dysbiotic (inflammatory) state, we established specific changes in specific microbiota that were significant by both Wilcoxon and two test methods (p <0.05) for each comparison by phase 3 study day. The indicated polymerase chain reaction (PCR) targets were each significantly higher in average relative abundance in the indicated groups relative to the comparator group*.

After only two daily vaginal applications of N9, statistically significant changes in the % RA of *Actinomyces spp* were observed when compared to the HEC placebo treatment (*p* < 0.05). These changes were not seen at later time points, suggesting this was an initial response to the presence of N9 and led to other changes with notable increases in *Haemophilus spp*. and *Helococcus spp*. on study D7. These were also significantly higher levels than observed in the Sham group. Most notably, the % RA of *Haemophilus spp*. in the N9-treated samples also proved to be significantly higher than in the BZK-treated animals on both D7 and D9, consistent with an overgrowth of this organism relative to the other community members specific to the N9 treatment. This increase resulted in the previously noted establishment of a novel CT dominated only by *Haemophilus spp*. Treatment with BZK created significant shifts in % RA for several bacterial targets that were more prevalent at later time points, suggesting an increasing shift with more doses of the irritant. After two doses (study D3) of BZK, there were significantly increased levels of *Acidovorax spp*. and *Corynebacterium spp*. relative to the HEC placebo control (*p* < 0.05). The significant increase in *Corynebacterium spp*. persisted on D7 and D9 (*p* < 0.05) relative to the HEC placebo-treated animals.

Like the impact of N9, BZK also created CTs that were associated with the treatment and not observed in the other groups. Evaluations of the change in % RA indicated that BZK enhanced the growth of *Corynebacterium spp.*, leading to overgrowth of this organism relative to the other treatment groups as well as the initial VMB community profiles for animals in the BZK group. The Cy CT was also created by the BZK treatment and appeared to be more frequent at later times in the study and persisted during the recovery period in contrast to the H CT that quickly resolved after cessation of the N9 application. The % RA analysis uncovered additional organisms that were represented as minor proportions of the total community and not clearly considered by the CT methodology. Specifically, treatment with BZK increased the levels of *Helcoccus spp*. and *Rhodococcus spp*. relative to the HEC group at later time points during treatment (Table 3). Finally, *Cloacibacterium spp*. also showed significant increases in the BZK-treated animals relative to the HEC and N9 treatments but only on D9.

### Vaginal Inflammation as an Indicator of VMB CT Health Contribution

**Figure 6:**
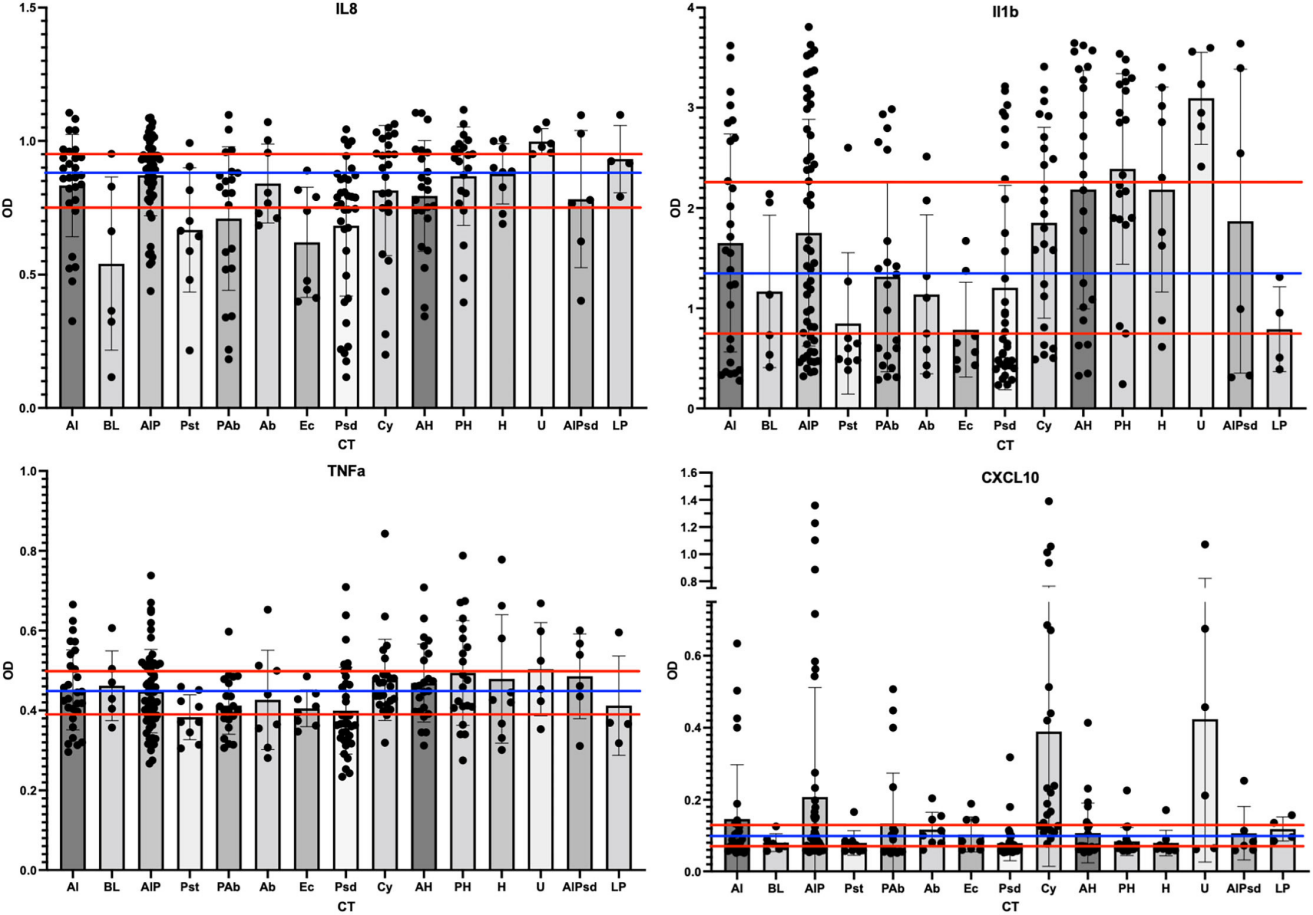
Scatter plots of the cytokine levels for each of the CT. The ELISA results for each phase 3 sample were grouped by assigned CT and then scatter-plotted. The bar shows average levels of the group with standard deviations shown as bar and whiskers. The tertile divisions (high, medium, and low were determined from all samples in the study) are shown as horizontal red lines, and binary divisions (high and low) are shown by the blue horizontal line. IL-8 outcomes are shown in the top left panel, IL-1b top right, TNF-a lower left, and CXCL10 lower right.

We automated previously reported customized ELISAs for selected ovine cytokines, namely, IL-1β, IL-6, IL-8, IL-17α, CXCL10 (aka IP-10), and TNF-α (28), to qualify the inflammation state of the vaginal environment associated with CTs. In addition to the 264 swab samples from the phase 3 study, 30 unrelated previously analyzed swab samples and a dilution series of synthetic standards were analyzed on each ELISA. The results from the IL17α and IL6 ELISAs indicated that these two proteins were rarely detected in the 264 samples from the phase 3 study (data not shown). As a result, these two targets were not considered useful for ovine vaginal evaluations, and no further analyses were completed. Of the four other targets, IL-8 was the most often detected (92% of samples). IL-1β was the next most common (76% of samples). For analyses, OD values without conversion were utilized to categorize each sample using tertile or binary divisions of the swab results (Figure 6; red and blue horizontal lines respectively). This approach supported the description of each sample as inflamed or normal (not inflamed) through a number of models ultimately leading to two distinct models (inflamed 1 and 4, abbreviated Infl1 and Infl4). The tertile Infl1 categorization resulted in the assignment of “inflamed” to 163 (62%) of the 264 samples, which appeared to be an overestimation of inflammation. The same model with a binary (high/low via 59^th^ percentile categorization) assignment showed 95/264 (36%) to be inflamed, which likely underestimated the status. Finally, the Infl4 model used only the IL-8 and IL-1β data (selected because of the greatest detection frequency) that with binary assessment identified 174/264 as inflamed (66%). Given the nature of the chemical irritants, we expected relatively high numbers of inflamed samples, so we completed subsequent data analyses with the binary model Infl4.

After the assignment of inflammation status, CTs were associated with marker organisms (Figure 5). After grouping the samples into the appropriate CT, the cytokine levels for each of the four useful targets were considered independently. Any sample with a high level was marked as inflamed for that cytokine and counted. When the majority of samples in a CT were found to associate with inflamed state for three of the four cytokine targets, the CT was marked as dysbiotic. For those where the majority was low inflammation levels, the CT was assigned as eubtioic. Through this clustering approach, several CTs were associated with an inflamed, dysbiotic state, helping to identify organisms predicted to indicate likely inflammation in the ovine vaginal vault (Figure 5, bottom). Conversely, a set of organisms were also identified that would predict eubiosis and non-inflamed status. *Pseudomonas, Bacteroides, Leptotrichia, E. coli*, and *Pasteurella* alone, or in combination with *Actinobacillus*, were found to be predictors of non-inflamed status. Interestingly, *Pasteurella*, in combination with *Haemophilus*, was predictive of inflammation. Finally, the dominance of *Ureaplasma* and *Corynebacterium* in VMB communities was a strong predictor of inflammation (Figure 5). In work outside of this manuscript, using these marker organisms, we associated their Gram-stain morphotypes to help direct the establishment of a scoring system for the Gram-stained slides (Vincent et al., under revision) and utilized diversity to support the counting of bacteria in specific Gram-stain and morphotype. Remarkably, the data suggested that the eubiotic ovine VMB communities would be dominated by Gram-negative organisms (e.g., *Pseudomonas* and *E. coli*), and that dysbiotic communities would include larger numbers of Gram-positives (e.g., *Corynebacterium*).

## Discussion

To our knowledge, this analysis was the first to comprehensively characterize VMB microbiota in the ovine vagina. Using our molecular approaches, CTs and biomarker organisms that could be associated with vaginal inflammation states were identified, as indicated by data from customized ELISAs for four relevant cytokines. Through the application of selected irritants, VMB community structure changes were noted after the use of N9 and BZK that were not seen in the Sham or control groups. However, because VMB in sheep had not previously been reported for drug testing, indicators and flora describing eubiosis and dysbiosis were not well-known in the sheep vaginal drug testing model. To complete foundational analyses, a total of 16 CTs were identified across two breeds of sheep housed under different conditions with access to different foods. Because the current data have clearly identified biomarker bacteria for each of the CT, changes in the absolute abundance of these marker organisms associated with treatments should indicate VMB shifts toward health or dysbiosis.

The quantification of the shGAPDH gene as a marker for cell numbers in each sample also proved useful for ensuring optimal sample collection and potentially showing impacts of vaginal applicants. However, the selected irritants in this study did not lead to dramatic impacts on the number of cells shed from the treated mucosa. Likewise, the 16S analyses allowed initial evaluations of the impact of irritants on general vaginal health. These control targets should be considered for individual training of staff before completing extensive preclinical evaluations of test articles. The results indicated that bacterial loads were decreased in the BZK and N9 groups initially with continued suppression during the BZK treatment and marked increases in bacterial load in the N9 treatment. Treatment with BZK and N9 induced shifts in VMB CT, such as unique CT, that were produced by the applicants. Both irritants altered CTs by enhancing the growth of selected organisms and suppression of others. Interestingly, the N9 treatment produced temporary impacts that resolved during the recovery period, while in the BZK treatment group, the changes persisted, leading to significant differences from the other recovery samples. Finally, the evaluation of % RA in both the N9 and BZK groups uncovered additional marker organisms (e.g., H, Cu, and U CT groups which had predominantly Haemophilus, Corynebacterium, and Ureaplasma) that were benefitted by the irritants. It remains to be determined if these organisms are associated with dysbiosis and inflammation; however, they were not common in the PD or Sham samples.

The ELISA analysis of cytokines from the sheep vaginal fluid swab samples showed that IL-17α and IL-6 were rarely seen in any of the sheep samples and, therefore, were subsequently deleted from the analysis. The four remaining cytokines, TNF-α, IL-8, IL1-β, and CXCL-10, were seen more commonly throughout the samples and were used to determine the presence or absence of inflammation in the samples. Although the custom ELISAs target a short list of cytokines, these particular proteins have clinical relevance for vaginal inflammation studies (7, 44, 45). These four targets were utilized as markers of inflammation by binary designation (values above the mean were marked as inflamed) with three of the four above the mean value, leading to the sample being categorized as inflamed. Such direct measures of vaginal health have been widely used in other preclinical and clinical study designs (1, 3, 7, 28, 44, 45).

Most importantly, these foundational studies provided clear VMB community structures for two breeds of sheep. The data are limited to these two breeds and to the lab animal housing approaches employed by the two geographic locations and research teams. Throughout the studies, great care was taken to limit the contamination of the sampling materials that were consistently confirmed to be free of bacterial genomes. The use of both NGS and qPCR approaches supported the accurate identification of community members and, despite differences to other reported VMB, provided an optimized method to ensure consistency across sampling teams, adding confidence that the observed CTs were accurately measured. Care should be taken in exploring the microbiota profiles from other breeds or housing and feed conditions, but the application of our workflow should support the accurate identification of alternate community structures. Similarly, additional studies are needed to fully understand the impacts of applicants on VMB CTs outside the 16 identified in this project. In parallel with this molecular study, we developed a more approachable scoring system based on Gram-staining of vaginal fluid that is reported separately (Vincent, et al.; submitted).

Characterization of the ovine VMB under these conditions was completed to help improve the utility of this model for preclinical testing of devices and applicants. The sheep vaginal environment offers many noted advantages over other animal models. However, the composition of VMB is not directly comparable to the Lactobacilli-dominated VMB seen in most women (1, 3–7, 13). In fact, no animal VMB community accurately models the specific composition of the human VMB but have been used as surrogates that are useful for indications of environmental perturbations, as shown by our application of the N9 and BZK irritants in the ovine model. Another distinction is the estrus cycle experienced by sheep rather than the menstrual cycle observed in humans. As previously described (20, 21, 24, 29), the ovine estrus cycle actually has noted similarities to the menstrual cycle with a rise in estradiol after stimulation by FSH from GnRH, an LH surge stimulating ovulation/estrus, and a rise in progesterone from the corpus luteum after ovulation. The additional qualities of the ovine vaginal vault, such as accommodation of human-size devices and applicant volumes, as well as greater similarity in epithelial thickness and anatomical structure, support the use of sheep as well as continued study of the vaginal environment to advance the utility of the model.

Finally, the current results indicated that repeated vaginal sampling had no discernable effect on the VMB or individual community members. Furthermore, the use of the VMB array provided useful data in a cost-effective and higher throughput fashion, adding the more accurate quantification provided by qPCR relative to NGS. The development of this type of array has been previously described (35, 36, 42), allowing for future refinement based on additional NGS data from other breeds of sheep or housing conditions. Importantly, as the ovine model is further utilized for the testing of vaginal applicants and devices, the datasets have confirmed the utility of the BZK and N9 test articles as irritants that differently impacted the VMB profile. These compounds can now be recommended as positive controls for irritation.

In summary, the datasets and associated analyses strongly support the utility of the ovine model for the testing of vaginal applicants and devices. To broaden the ability of the model system to predict toxicity or safety issues, the evaluation of VMB samples is strongly supported by these results, but additional studies are needed to fully integrate the results with health status.

## Data Availability Statement

The datasets presented in this study can be found in online repositories. The names of the repository/repositories and accession number(s) can be found in the article/Supplementary Material.

## Ethics Statement

The animal study was reviewed and approved by Sinclair Research Center Animal Care and Use Committee and the University of Texas Medical Branch Animal Care and Use Committee.

## Author Contributions

All the authors contributed to the final text and agreed to its publication. Preparation of the data and analyses and manuscript drafts were completed by RP, NR-H, and KV. AM, CM, GM, LD, and TM produced the sheep materials and performed the analyses of the vaginal fluids. RP, AM, and CM created and analyzed the VMB data and CT designations. Study oversight and planning were completed by RP, GS, CO'N, CW, TM, and KV. RP, NR-H, GM, TM, MM, GV, and KV were responsible for the final study designs. All authors contributed to the article and approved the submitted version.

## Funding

This study was supported through the Comprehensive Resources for HIV Microbicides and Biomedical Prevention Contract Program in the Division of AIDS at NIAID/NIH (Contracts HHSN272201000001C and HHSN272201600008I). Funding from R01AI112015 and U19AI11304803 from the National Institute of Allergy and Infectious Diseases provided partial support for the initial studies. The content is solely the responsibility of the authors and does not necessarily represent the official views of the National Institutes of Health.

## Conflict of Interest

NR-H was employed by Alpha StatConsult, LLC. GS, CO'N, and CW were employed by Advanced Bioscience Laboratories, Inc. RP and KV were paid consultants to Advanced Bioscience Laboratories, Inc. TM was employed by Sinclair Research Center. The remaining authors declare that the research was conducted in the absence of any commercial or financial relationships that could be construed as a potential conflict of interest.

## Publisher's Note

All claims expressed in this article are solely those of the authors and do not necessarily represent those of their affiliated organizations, or those of the publisher, the editors and the reviewers. Any product that may be evaluated in this article, or claim that may be made by its manufacturer, is not guaranteed or endorsed by the publisher.

## Supplementary Material

Supplemental Figure 1 Average longitudinal shGAPDH and 16S copies detected by study day in each of the four groups of phase 3 animals. The average number of shGAPDH (left) or 16S bacterial rDNA (right) quantified by quantitative polymerase chain reaction (qPCR) are shown over time. SDs are plotted. Sham animals are shown in black, hydroxyethylcellulose (HEC)- treated in red, nonoxynol-9 (N9)-treated in green, and benzalkonium chloride (BZK)-treated shown in blue.
