# Supplementary figures and images for: Characterization of the Ovine Vaginal Microbiome and Inflammation Patterns as an Improved Testing Model of Human Vaginal Irritation
Source: Front Reprod Health. 2021 Dec 7;3:714829. doi: 10.3389/frph.2021.714829 (PMC9580801; doi:10.3389/frph.2021.714829)

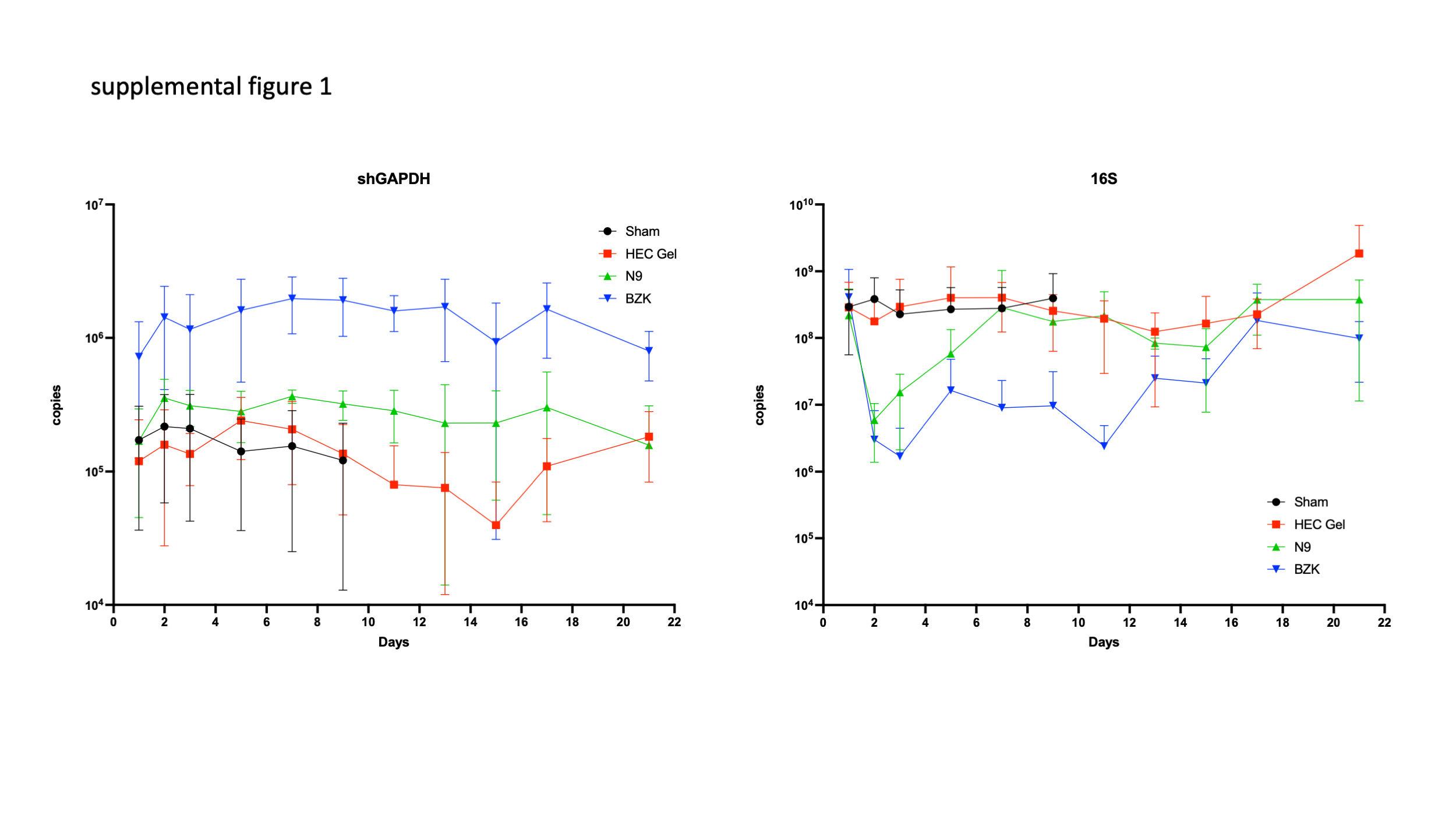

Supplement: Supplemental Figure 1 — Average longitudinal shGAPDH and 16S copies detected by study day in each of the four groups of phase 3 animals. The average number of shGAPDH (left) or 16S bacterial rDNA (right) quantified by quantitative polymerase chain reaction (qPCR) are shown over time. SDs are plotted. Sham animals are shown in black, hydroxyethylcellulose (HEC)- treated in red, nonoxynol-9 (N9)-treated in green, and benzalkonium chloride (BZK)-treated shown in blue. [file Image_1.TIFF]
